# Vitamin D in health and disease: Current perspectives

**DOI:** 10.1186/1475-2891-9-65

**Published:** 2010-12-08

**Authors:** Ran Zhang, Declan P Naughton

**Affiliations:** 1 AllergyMatters Ltd, 5a Kingston House Estate, Portsmouth Rd., Long Ditton Surrey, England KT6 5QG, UK; 2 School of Life Sciences, Kingston University, Penrhyn Road, Kingston upon Thames, London KT1 2EE, UK

## Abstract

Despite the numerous reports of the association of vitamin D with a spectrum of development, disease treatment and health maintenance, vitamin D deficiency is common. Originating in part from the diet but with a key source resulting from transformation by exposure to sunshine, a great deal of the population suffers from vitamin D deficiency especially during winter months. It is linked to the treatment and pathogenesis and/or progression of several disorders including cancer, hypertension, multiple sclerosis, rheumatoid arthritis, osteoporosis, muscle weakness and diabetes. This widespread deficiency of Vitamin D merits consideration of widespread policies including increasing awareness among the public and healthcare professionals.

## 1. Introduction

Vitamin D is a group of fat-soluble prohormones which were identified after the discovery of the anti-rachitic effect of cod liver oil in the early part of the 20^th ^century. The vitamin found in cod liver oil was designated "D" following Vitamin A, B and C, which had been discovered earlier [1]. The two major biologically inert precursors of vitamin D are vitamin D3 (cholecalciferol) and vitamin D2 (ergocalciferol) [2,3]. Vitamin D3 is formed when 7-dehydrocholesterol in the skin is exposed to solar ultraviolet B (UVB, 290-320 nm), and then converted to previtamin D3. In a heat-dependent process, previtamin D3 is immediately converted to vitamin D. Excess UVB rays transform previtamin D3 into biologically inactive metabolites, tachysterol and lumisterol. Vitamin D2 is plant derived, produced exogenously by irradiation of ergosterol, and enters the circulation through diet [1].

**Figure 1:**
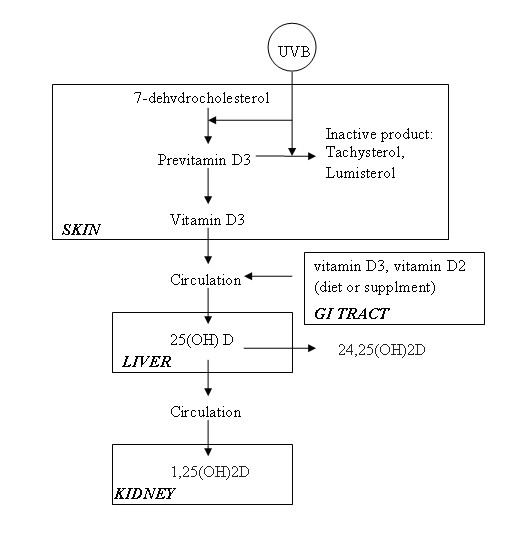
**Sources, sites, and processing of vitamin D metabolites**.

Both vitamin D precursors resulting from exposure to the sunshine and the diet are converted to 25-hydroxyvitamin D [25(OH)D] (calcidiol) when they enter the liver [4]. 25(OH)D is the major circulating form of vitamin D and is used to determine vitamin D status. In order to be biologically active, additional hydroxylation in the kidneys is needed to form active 1,25-dihydroxyvitamin D [1,25(OH)2D] (calcitriol) [5]. The process of vitamin D formation is summarized in Figure 1. Humans obtain vitamin D through dietary intake and exposure to sunlight. Very few foods naturally contain vitamin D. Oily fish such as salmon, mackerel, and sardines are rich in vitamin D3. Egg yolks are reported to contain vitamin D though the amounts are highly variable. Moreover, the cholesterol content of egg yolks makes it a poor source of vitamin D. Also, a small number of foods are fortified with vitamin D such as milk, orange juice and some bread and cereals [6,7]. A list of vitamin D content in different food sources is shown in Table 1.

**Table 1:** Vitamin D3 and D2 sources and content*

Source		Typical Vitamin D content
Natural source	Salmon, fresh, wild (3.5 oz)	600-1000 IU of vitamin D3
	
	Salmon, fresh, farmed (3.5 oz)	100-250 IU of vitamin D3 or D2
	
	Salmon, canned (3.5 oz)	300-600 IU of vitamin D3
	
	Sardines, canned (3.5 oz)	300 IU of vitamin D3
	
	Mackerel, canned (3.5 oz)	250 IU of vitamin D3
	
	Tuna, canned (3.6 oz)	230 IU of vitamin D3
	
	Cod liver oil (1 tsp)	400-1000 IU of vitamin D3
	
	Shiitake mushrooms, fresh (3.5 oz)	100 IU of vitamin D2
	
	Shiitake mushroom, sun-dried (3.5 oz)	1600 IU of vitamin D2
	
	Egg yolk	20 IU of vitamin D3 or D2
	
	Exposure to sunlight, UVB (0.5 MED^†^)	3000 IU of vitamin D3

Fortified foods	Fortified butter	50 IU/3.5 oz, usually vitamin D3
	
	Fortified milk	100 IU/8 oz, usually vitamin D3
	
	Fortified orange juice	100 IU/8 oz, vitamin D3
	
	Fortified yogurts	100 IU/8 oz, usually vitamin D3
	
	Infant formulas	100 IU/8 oz, vitamin D3
	
	Fortified margarine	430 IU/3.5 oz, usually vitamin D3
	
	Fortified cheeses	100 IU/3 oz, usually vitamin D3
	
	Fortified breakfast cereals	100 IU/serving, usually vitamin D3

* IU refers to international units, which equal 25 ng.

† About 0.5 MED of UVB radiation would be absorbed after an average of 5 to 10 minutes of exposure (depending on the time of day, season, latitude, and skin sensitivity) of the arms and legs to direct sunlight.

Vitamin D plays an important role in maintaining an adequate level of serum calcium and phosphorus. Without vitamin D, only 10 to 15% of dietary calcium and about 60% of phosphorus is absorbed [8-10]. Therefore vitamin D has a great effect in forming and maintaining strong bones. It has also recently been found that vitamin D receptors exist in a variety of cells thus it has a biological effect on more than mineral metabolism. The aim of this report is to review key aspects relating to vitamin D deficiency, its causes, and studies on prevention of and treatment of major conditions/diseases. Thus, following a general literature review on deficiency and its causes, an overview of major meta-analyses of Vitamin D supplementation is given. This systematic approach covers meta-analyses listed in Pubmed during the past 2 decades.

## 2. Vitamin D deficiency

### Vitamin D deficiency and intoxication

Vitamin D deficiency occurs when people do not have an appropriate dietary intake or exposure to UVB rays. It is universally accepted that the circulating level of 25-hydroxyvitamin D should be used as an indicator of vitamin D status due to its ease of measurement, long half-life in circulation (approximately 2 or 3 weeks), and the correlation of its level with clinical disease states [1,11,12]. Although no consensus on an optimal level of 25-hydroxyvitamin D has been reached, vitamin D deficiency is defined by most experts as a level of less than 20 ng per millilitre (50 nmol per litre) [13-16]. A level of 25-hydroxyvitamin D of 21 to 29 ng per millilitre (52 to 72 nmol per litre) is considered as an insufficiency of vitamin D, and sufficient vitamin D should reach a level of 30 ng per millilitre or greater [17].

In 1997, the Institute of Medicine of the US National Academy of Sciences recommended new adequate intakes for vitamin D as 200 IU for children and adults up to 50 years of age, 400 IU for adults 51 to 70 years of age, and 600 IU for adults 71 years of age or older [18]. However, a great number of studies revealed that without adequate sun exposure, children and adults require approximately 800 to 1000 IU per day [19-22].

Vitamin D intoxication is extremely rare. Studies showed that doses of more than 50,000 IU per day, which raises 25-hydroxyvitamin D to more than 150 ng/ml, is associated with hypercalcemia and hyperphosphatemia [8,9,23]. Even doses of 10,000 IU of vitamin D3 per day for up to 5 months did not cause toxicity [24]. However, patients with chronic granulomatous disorders should be cautious with the dose of vitamin D since macrophage production of 1,25-dihydroxyvitamin D causes hypercalcemia and hyperphosphatemia [8,9,23].

#### *2.1 *Causes of vitamin D deficiency

There are many causes of vitamin D deficiency. Generally, they can be divided into two groups: UVB-related deficiency and medical/physical condition-related deficiency.

##### 2.1.2 UVB-related deficiency

##### The elderly

The elderly, due to the decreased presence of skin 7-dehydrocholesterol which is the precursor for UVB mediated synthesis of vitamin D, are particularly at risk of vitamin D deficiency. Moreover, reduced mobility or institutionalization that discourages sun exposure, reduced renal production of 1,25-dihydroxyvitamin D as well as decreased intake of fortified foods pose great difficulties in vitamin D formation in body [25,26].

##### Dark skin

People with dark skin have great amounts of melanin in their epidermis. Melanin competes with 7-dehydrocholesterol for absorption of UVB photons. Therefore, people of color are less efficient in producing vitamin D than are whites. It is reported that a person with skin type 5/6 (dark skin) requires 10-50 times the exposure to sunlight to produce the same amount of vitamin D as does a white person with skin type 2/3 [27].

##### Season, latitude, and the time of day

It has been established that the ozone layer can absorb UVB radiation above 290 nm which is responsible for generating previtamin D3. Zenith angle, defined as the angle of the sunlight reaching the Earth's surface, decides the thickness of ozone layer which sunlight needs to penetrate. The thicker the ozone layer is, the fewer amounts of UVB photons can reach the earth, thus few previtamin D3 can be produced. Zenith angle is dependent on factors such as time of day, season of the year, and latitude. Thus those factors have great effects on vitamin D production [28,29]. For example, residents of Boston (42°N), Edmonton, Canada (52°N) and Bergen, Norway (61°N) can not produce sufficient quantities of vitamin D in their skin for 4, 5, and 6 months, respectively [6].

##### Sunscreen users

Sunscreens can efficiently absorb UVB radiation. This dramatically prevents the interaction of UVB with 7-dehydrocholesterol, the process of previtamin D3 generation. It has been shown that when used properly, a sunscreen with a sun protection factor of 8 reduces the production of previtamin D3 by 95%, and 99% by a sun protection factor of 15 [30,31].

#### 2.1.2 Medical/physical condition-related deficiency

##### Fat malabsorption

As a fat-soluble vitamin, vitamin D requires the presence of dietary fat in the gut for absorption. Certain pathological conditions, such as Crohn's disease, cystic fibrosis (CF), celiac disease, surgical removal of part of the stomach or intestines are associated with fat malabsorption and thus may lead to vitamin D deficiency. For example, CF patients suffer from pancreatic exocrine insufficiency. This results in malabsorption of fat-soluble vitamins, including vitamin D. CF patients, depending on the degree of exocrine insufficiency, absorb approximately 50% less vitamin D than normal [32].

##### Anticovulsant use

Anticonvulsants, also called antiepileptic drugs, have been used to treat epileptic seizures and bipolar disorder. It is well recognized that long-term use of some antiepileptic drugs, including phenobarbital, phenytoin, and carbamazepine and the antimicrobial agent rifampicin (RIF) can result in osteomalacia [33-37]. The induction of the catabolism of 1,25-dihydroxyvitamin D by these drugs is thought to contribute to their deleterious side effects.

##### Chronic kidney disease

In order to become biological active vitamin D, kidney plays an important role in this transforming process. Chronic kidney disease such as patients with stage 4 or 5 chronic kidney disease, as well as those requiring dialysis, leads to an inability to make sufficient 1,25-dihydroxyvitamin D which has a direct effect in inhibiting parathyroid hormones expression [38,39]. Thus 1,25-dihydroxyvitamin D3 intake is needed to maintain calcium level in blood as well as to control parathyroid hormone levels.

##### Obesity

It has been known for a long time that obese people are prone to be vitamin D deficient since they have lower 25-hydroxyvitamin D levels [40-43]. A number of studies proved that the vitamin D3 precursor 7-dehydrocholesterol levels in the skin of obese people were not significantly different from non-obese people [26,44]. One explanation was that the subcutaneous fat, which is known to store vitamin D, sequestered more of the cutaneous synthesized vitamin D, which results in less release of vitamin D from the skin into the circulation in the obese subject than non-obese subject [45].

### Epidemiology of vitamin D inadequacy

Several studies showed that 40 to 100% of U.S. and European elderly men and women still living in the community (not nursing homes) are deficient in vitamin D [13-15]. It has already become a largely unrecognized global epidemic. Vitamin D inadequacy can be seen in young adults as well as healthy children. For example, 48% of white preadolescent girls in a study in Maine [46] and 52% of Hispanic and black adolescents in a study in Boston are vitamin D deficient [47]. In Europe, where very few foods are fortified with vitamin D, children and adults would appear to be at especially high risk [48-50]. A study of middle aged British adults showed that 60% are vitamin D insufficient, and the number rose to 90% during winter and spring [51].

## 3. UVB in vitamin D formation

The resurgence of vitamin D deficiency has been attributed to the lack of exposure to sunlight that has been a growing concern in the past few decades owing to associations with skin cancer. From this standpoint, along with the modern predominance for indoor living and working, it is apposite to look at photo-induced vitamin D activation. In future we may replace artificial light with a form that is more efficient at meeting our requirements for vitamin D. Ultraviolet (UV) rays are electromagnetic waves with wavelength of between 400 nm and 10 nm. UV can be divided into three components according to wavelength: UVA (320-400 nm), UVB (290-320 nm), and UVC (100-290 nm). They have different skin penetration abilities, and generate different biological effects.

UVB plays a key role in vitamin D formation. It is absorbed by the epidermal layer, where the highest concentration of 7-dehydrocholesterol exists. It has been found that the optimum wavelength range for the production of vitamin D is between 295 and 300 nm. This narrow range is sometimes referred to as D-UV [52]. As vitamin D is rare in the diet consumed by human beings, the major source of vitamin D is exposure to sunlight, particularly the UVB component [23,53,54]. Another advantage of UVB exposure in formation of vitamin D is that UVB exposure does not result in excessive production of vitamin D, which causes risks of intoxication. This can be explained that the previtamin D3 that is formed and the thermal isomerization product vitamin D3 that does not go into the circulation absorb UVB radiation and isomerize to several photoproducts which have little activity on calcium metabolism [55].

### Choice of wavelength

As UVB exposure is not associated with vitamin D intoxication, the major concern of using UVB to boost vitamin D content is the erythema reaction, which is a result of cell irritation and destruction caused by ultraviolet radiation. In the UVB region, the wavelength range between 290 and 297 nm has the greatest erythema effect on the human body with a steep decrease above 297 nm. Therefore UVB tubes with spectral features of minimal irradiance from 290 to 297 nm should always be chosen to treat vitamin D deficiency.

### The duration of exposure

The duration of exposure determines the dose of UVB one receives. The product of the UVB lamp power output (mW/cm^2^) and the duration of exposure is UVB radiation (mJ/cm^2^). Minimal erythema dose (MED) is defined as the minimum amount of UVB radiation that produces redness 24 hours after exposure. It is used when using UVB to treat psoriasis in order to minimize the potential for developing erythema. Therefore the radiation should not be more than one MED for safety considerations when treating vitamin D deficiency. Thus, a limit should be put on exposure time depending on power output.

It should be noted that MED varies with skin type (Table 2) and there are large variations in MED even within the same skin type [56]. According to the U.S. Food and Drug Administration and the American Academy of Dermatology, there are six skin-type categories: skin type I-VI. Generally the darker the skin, the harder it is for the skin to burn, thus the higher the value of MED which results in a longer exposure duration to achieve a certain value of MED.

**Table 2:** Skin type categories from Food and Drug Administration (FDA) and the American Academy of Dermatology.

Skin types	Sun history	Example
I	Always burns easily, never tans, extremely sensitive skin	Red-headed, freckled, Celtic, Irish-Scots

II	Always burns easily, tans minimally, very sensitive skin	Fair-skinned, fair-haired, blue-eyed Caucasians

III	Sometimes burns, tans gradually to light brown, sun-sensitive skin	Average-skinned Caucasians, light-skinned Asians

IV	Burns minimally, always tans to moderate brown, minimally sun-sensitive	Mediterranean-type Caucasians

V	Rarely burns, tans well, sun-insensitive skin	Middle Easterners, some Hispanics, some African-Americans

VI	Never burns, deeply pigmented, sun-insensitive skin	African-Americans

#### 3.1 Vitamin D production

It has been reported that exposure of 6-10% of the body surface to 1 MED is equivalent to ingesting about 600-1000 IU of vitamin D [57]. In general, when using UVB radiation in vitamin D formation, the factors influencing vitamin D production can be summarized as: the UVB radiation, the exposure duration and body exposure area. The stronger the radiation is (under certain limits), the longer the exposure duration is (under certain limits), the larger the body exposure area is, the more vitamins can be produced.

A number of studies have been conducted to improve vitamin D content using UVB rays [58-61]. 45 female psychogeriatric patients in nursing homes received 1/2 MED UVB irradiation, three times per week, for 12 weeks. Results showed that 1,25-dihydroxyvitamin D levels increased significantly from 30 nmol/l to 60 nmol/l on average [62]. Another study using UV light to treat vitamin D deficiency resulted from malabsorption indicated that 25-hydroxyvitamin levels increased by 28% at the end of 8 weeks [63].

#### 3.2 Contraindications

UVB irradiation does not appear to suit some individuals who develop headache, nausea and possibly vomiting and rise of temperature after exposure. Also people with sensitive skin may react strongly to UV rays, thus are unsuitable for UV treatment [64].

Certain drugs could make individuals become more susceptible than normal to the effects of UV radiation, examples being gold, the sulphonamides, insulin, thyroid extract, and quinine. Therefore UVB should not be used in conjunction with these drugs. An area which has recently been subjected to doses to X-rays is not suitable for UVB treatment since it may cause carcinoma of the skin. UV may also cause the aggravation of certain diseases such as pulmonary tuberculosis, acute eczema or dermatitis [64]. Thus it is not appropriate to apply UVB in these conditions.

#### 3.3 Vitamin D intake in different conditions

The association of low status of vitamin D with several diseases has been long established. Increasing vitamin D levels through oral supplementation or sunlight exposure is vital in the management of these conditions. Also, vitamin D has been used to treat some minor abnormalities, such as tooth loss and depressed moods, in order to maintain a healthy lifestyle. Moreover, research during the past two decades has highlighted the important role of vitamin D in reducing the risks of a series of conditions including cancer, multiple sclerosis, hypertension, to name a few. In this section, the utility of vitamin D for different purposes such as disease management, prevention, and improvement of quality of life will be discussed.

## 4. Disease management

A large number of trials have been conducted over the past two decades into the effects of vitamin D on disease management and prevention. The efficacies of vitamin D, as demonstrated by the meta-analyses reported in this period, are largely unconvincing apart from in relation to falls and fractures in the elderly. These meta-analyses, summarized in Table 3 do not show a substantial role for vitamin D either through prevention or treatment of many conditions for which it is associated. It should be noted that many of the meta-analysis relied on disparate studies which introduces significant questions regarding the results. Despite this overview, and in a somewhat unorthodox approach some key studies will be discussed below that may lead to more focused and appropriate trials.

**Table 3:** Systematic reviews on Vitamin D for prevention or treatment (in chronological order)

Area	Number of studies	Type	Doses	Conclusion	Reference
Cardiovascular events	17	Prospective	~1000IU	S not SS	162

Cardiometabolic outcomes	31	Observ/Trial	Range	No effect	163

Fracture prevention	7	Randomised	10-20 mcg + Ca	No effect Reduced risk	164 164

Physical performance	8 8	Observational Intervention	- -	Positive (5/8) Mixed	165 165

Kidney disease (-dialysis)	16	RCT	-	Uncertain	166

Kidney disease (+dialysis)	60	RCT	-	Uncertain	167

Cystic fibrosis	3	(q)RCT	800-1600IU	No effect	168

Risk of falling	8	RCT	700-1000IU 200-600IU	Reduced risk No effect	169 169

Hypertension	11	RCT	-	S - SS	170

Risk of falling	111*	RT or interv.	-	No effect	171

Fracture prevention	45	(q)RCT	- +Ca	No effect S not SS	172 172

Risk of Type 1 diabetes	5	Observational	-	Uncertain	136

Parathyroid hormone	52	Intervention	_	Decrease	173

Mortality	18	RCT	2300-2000IU	Uncertain	174

Risk of fall/fracture	9	-	-	Decrease (trend)	175

Risk of fracture	12	RCT	700-800IU 400IU	Decrease (trend) No effect	176 176

Risk of fall	10	RCT	-	Decrease	177

Bone density Fracture	17 17	RCT RCT	- -	Uncertain Reduction	178

S not SS: Small effect - not statistically significant (sub-groups are discounted); RCT: Randomised controlled trial {(q) - quasi}; * Multiple factor study.

### Osteoporosis

Approximately 33% of women aged between 60 to 70 and 66% of those over 80 have osteoporosis [13]. The link between vitamin D deficiency and osteoporosis has been well established especially in the elderly. Vitamin D deficiency is associated with the marked suppression in intestinal Ca absorption and the impairment of Ca balance, which results in low bone mineral content and density. Reduced bone mineral density (BMD) increases the risk of fractures, which significantly contributes to morbidity and mortality of older persons [65,66]. Hip fracture is expected to be more prevalent worldwide with the increase in aging of the population, and the consequences are severe: 50% of older persons have permanent functional disabilities, 15% to 25% require long-term nursing home care, and 10% to 20% die within 1 year.

The efficacy of vitamin D as a prevention of fracture has been demonstrated by a number of studies. Trials using 700 to 800 IU/d oral vitamin D with or without calcium supplementation found a significant 26% reduction in risk of sustaining a hip fracture and a significant 23% reduction in risk of sustaining any non-vertebral fracture versus calcium or placebo [67]. Also, a 3-year French study of 3270 elderly women (mean age 84 years) living in care home, where vitamin D deficiency is prevalent, indicates that calcium (1200 mg daily) and vitamin D (800 IU daily) can reduce the probability of hip and all non-vertebral fractures by 43% and 32%, respectively, compared with placebo [68,69]. Conversely, a Dutch study of 2578 healthy elderly women treated with a high calcium intake, daily supplementation with vitamin D 400 IU over 3.5 years showed no effect on the risk of hip fracture [70]. Therefore, whether 400 IU vitamin D is enough to prevent fracture in healthy elderly subjects with normal BMD needs further investigation.

### Muscle weakness

Muscle weakness is also a prominent feature of vitamin D deficiency. Patients with nonspecific muscle weakness, muscle aches and pains have also been found with vitamin D inadequacy [71,72]. Impaired muscle function is known to cause an increased number of falls which can lead to hip fractures. The incidence of falls go up substantially in those older than 70 years of age, and over 90% of hip factures resulted from a fall. It has been established that skeletal muscle tissue contains vitamin D receptor and may require vitamin D to realize maximum function [23,73].

It has been reported that increased vitamin D levels can improve muscle performance, and thus reduce the incidence of fall. In a 5-month randomized controlled trial, elderly people in a nursing home received 800 IU of vitamin D2 plus calcium daily, exhibited a 72% reduction in the risk of falls as compared with the placebo group [74]. Long-term intake of 700 IU vitamin D plus 500 mg of calcium in 246 community-dwelling older women also showed a beneficial reduction falls: the odds of falling declined by 46% compared with the placebo group [74]. However, the results from one trial suggested that 400 IU vitamin per day may not be clinical effective in preventing falls in the elderly [75].

### Hypertension

Millions of people are affected by hypertension worldwide. Growing evidence in recent years suggests that vitamin D has an important association with blood pressure. Animal experiments implicate 1,25-dihydroxyvitamin D in inhibiting renin expression in the juxtaglomerular apparatus and blocks proliferation of vascular smooth muscle cells (VSMC), which could influence systemic blood pressure [76-79]. Studies showed that Afro-Americans have a significantly higher prevalence of diastolic hypertension [80] and have lower 25-hydroxyvitamin D levels [81] compared with white Americans.

Reduced blood pressure has been found in people taking oral supplementation of vitamin D. In humans, skin exposure to UVB, which is the major source of vitamin D formation, has been linked with lower blood pressure [82-84]. An 8-week single intervention study of 148 vitamin D deficient elderly women demonstrated a 9% decrease in systolic blood pressure with supplemental vitamin D (800 IU) plus calcium (1200 mg) compared with calcium alone [85]. In another study, patients with hypertension were exposed to UVB radiation three times a week for 3 months. Results showed that 25-hydroxyvitamin D levels increased by approximately 180%, and both systolic and diastolic blood pressure reduced by 6 mm Hg [84]. In contrast, a large prospective study of men and women found no association between intake of vitamin D from diet and supplements and hypertension incidents [86]. This difference may be attributed to the fact that the large observational study included subjects from the general public while other studies recruited hypertension patients. Thus the original vitamin D levels could have certain impacts on treatment effect.

### Multiple sclerosis

Multiple sclerosis (MS) is an autoimmune disease in which the body's immune system attacks myelin, a key substance that serves as a nerve insulator and helps in the transmission of nerve signals. It has been long recognised that MS is more common in temperate climates than the tropics [87,88], and annual and winter hours of sunlight have been proved to have the strongest negative correlation with the prevalence of MS [89,90]. One explanation is that the increase of vitamin D results from sunlight exerting a protective effect [91-93]. Studies also found that individuals with MS tend to have insufficient vitamin D levels [71,94-97].

However, only a few reports are available on the use of vitamin D in treating MS patients. One study (6 months) of the cytokine profile in MS patients with vitamin D (1000 IU/day) and calcium (800 mg/day) showed that 25-hydroxyvitamin D significantly increased from 42.5 ± 15 to 70 ± 20 nmol/l. Also vitamin D supplementation significantly increase serum transforming growth factor (TGF)-β1, which has been shown to be an important anti-inflammatory cytokine in animal models of MS [98]. The increased TGF-β1 suggests that vitamin D supplementation could potentially improve the symptoms of MS patients. Nonetheless, more studies are needed to establish vitamin D's efficacy in alleviating MS.

Apart from improving MS patients' health conditions, vitamin D has also been shown to have effects in preventing MS. One study revealed that women who used supplemental vitamin D (> 400 IU/day) had a 40% lower risk of MS than women who did not use vitamin D [99]. Another report among 7 million US military personnel reported that risk of MS decreased with increasing 25-hydroxyvitamin D which provided an evidence of vitamin D's protective role in MS developing [100].

### Malabsorption

As explained above, certain conditions cause malabsorption, which results in vitamin D deficiency. Low BMD can be frequently found in these conditions. Thus vitamin D supplementation is required to boost vitamin D sera levels. For example, Cystic fibrosis (CF) patients have inefficient vitamin D absorption due to pancreatic exocrine insufficiency. It is reported that 95% of a treated cohort of CF patients required 1800 IU (45 μg/d or 0.13 mmol) of ergocalciferol (vitamin D2) daily to achieve a 25OHD concentration above 25 ng/ml [101]. Vitamin D deficiency is prevalent in Crohn's Disease (CD). CD is an inflammatory bowel disease which often leads to osteopenia and osteoporosis due to malabsorption of vitamin D. One study conducted on 154 CD patients for 76 days showed that daily calcium (500 mg) and vitamin D (400 IU) supplementation was associated with an increase in bone mineral density [102].

## 5. Vitamin D associations with disease prevention

### Rickets

With the re-arrival of widespread vitamin D deficiency, the re-emergence of rickets, a scourge from the ninetieth century was inevitable. In a recent review, the deficiency rates found in countries where extreme sunlight levels are readily available but people are covered or avoid sunlight and do not have access to vitamin D-fortified foods [23]. Insufficient levels of vitamin D in breastfeeding mothers can, often unknowingly, lead to deficiencies in children. It is suggested that a supplement level of 400IU/d for infants as practiced in Canada is optimal [23].

### Cancer

The first study indicating that sunlight exposure may lower the risk of cancer was first made almost seven decades ago [103]. Garland and Garland were the first to propose that vitamin D deficiency may contribute to a higher risk of colon cancer mortality since vitamin D is formed in the skin through solar UVB radiation. More recently, the discovery of increased risks of certain types of cancer in those who are vitamin D deficient, suggests that vitamin D deficiency may account for thousands of premature deaths from colon [104], breast [105,106], ovarian [107], and prostate cancer [108] every year.

Vitamin D is one of the most potent hormones for regulating cell growth. It was discovered that many cell types contain vitamin D receptors. These receptors can be activated by 1,25(OH)2 D, and induce differentiation into normally functioning cells, and inhibit proliferation, invasiveness, angiogenesis, and metastatic potential. In tumor models such as cancers of the lung [109-111], colon [112], kidney [113], breast [114], and prostate [115], vitamin D played a role in activity against metastasis [116-120].

The protective relationship between sufficient vitamin D status and lower risk of cancer has been found in many studies. It has been reported that breast and colorectal cancer can be reduced by 50% with the concentration of 25-hydroxyvitamin D being >32 ng/mL [121,122]. A similar study of colorectal cancer found that a level of 34 ng/mL 25-hydroxyvitamin D can reduce the incidence by half, and 46 ng/mL can decrease it by two thirds [123]. A 4-year trial on postmenopausal women showed that 1100 IU/day vitamin D plus 1400-1500 mg/day calcium can substantially reduces all-cancer risk [121].

### Rheumatoid Arthritis (RA)

RA is an autoimmune disorder of unknown etiology in which both genetic and nongenetic factors contribute to disease susceptibility [124]. The immunomodulatory effect of Vitamin D has received increasing attention in recent years. Studies showed that when confronted by an inappropriate and overly exuberant immune response, vitamin D may act in a paracrine manner to decrease T cell responsiveness through the inhibition of cellular proliferation and reduction in lymphokine production [125-128]. Therefore vitamin D has a beneficial effect as an immunosuppressant.

Vitamin D has shown its ability to suppress the development of autoimmunity in animal experiments. For example, murine models of human RA demonstrated that when treated with active vitamin D, both the incidence and severity of the disease in mice decreased [129]. However the association of vitamin D intake and RA incidence in humans has not been well studied. One study of 30,000 women aged 55 to 69 over 11 years observed an inverse association between greater intake of vitamin D and RA risk [130]. Interestingly, vitamin D from supplements showed a stronger influence in RA development than did dietary vitamin D. However, further studies are needed to establish the effect of vitamin D in the prevention of RA.

### Diabetes

A diabetes epidemic has emerged during the 20^th ^century. The prevalence of diabetes for all age groups was estimated to be 2.8% in 2000, and this number will increase to 4.4% by 2030 [131]. It has been reported as early as the 1980 s that vitamin D deficiency inhibits pancreatic secretion and turnover of insulin, resulting in impaired glucose tolerance [132,133]. An association was found between low UVB irradiance, indicating a low level of vitamin D, and high incidence of type 1 diabetes, whereas the incidence rate approached zero in regions with high UVB irradiance [134-137].

A study of 83,779 women with no history of diabetes over 2-4 years showed that a combined daily intake of >1200 mg calcium and >800 IU vitamin D was associated with 33% lower risk of type 2 diabetes as compared with a daily intake of less than 600 mg of calcium and less than 400 IU of vitamin D [138]. Another study on 10,366 children in Finland over 31 years indicated that 2000 IU of vitamin D per day during their first year of life can reduce the risk of type 1 diabetes by approximately 80% [139].

### Tuberculosis (TB)

TB is a major global problem and responsible for 2 million deaths a year. It is estimated that one-third of the global population has latent TB infection [140], which poses great potential risks of reactivation in the future. In fact, before antibiotics came in to use, high doses of vitamin D were widely used to treat active TB [141]. Cross-sectional studies showed that patients with TB have lower 25(OH)D levels in comparison with control subjects [142,143]. Recently, it was found that low vitamin D status resulting from a vegetarian diet is an independent risk factor for active TB in South Asians [144].

Until now studies evaluating the effect of vitamin D on the body's immunity to mycobacteria, the family of bacteria that cause TB, are scanty. One study measuring 192 healthy adult TB contacts' showed that a single oral dose of 2.5 mg vitamin D significantly enhanced participants' whole blood ability to restrict functional whole blood (BCG-lux) luminescence in vitro [145]. It concluded that vitamin D enhances TB contacts' immunity to mycobacteria. However, the effect of vitamin D supplementation on TB incidence rates among deficient population with high rates of latent TB infection should be investigated to determine vitamin D's prevention effect in TB.

## 6. Health maintenance and Vitamin D

Besides treating and preventing a range of disease, vitamin D has also been applied in managing minor abnormalities so as to improve quality of life.

Periodontal disease is a common chronic inflammatory disease characterized by loss of periodontal attachment. It is the leading cause of tooth loss [146-150] which has a great impact in nutrient intake the quality of life [151-153]. Several epidemiologic studies revealed that there is link between osteoporosis and tooth loss, which indicate that the cause of osteoporosis could also contribute to periodontal disease. A 3-year study showed that supplementation with vitamin D (700 IU/d) plus calcium (500 mg/d) significantly reduced tooth loss in older people [154].

Mood changes with season, a common phenomenon is that anxiety and depression increase during winter months. An extreme case of this seasonality is a clinical syndrome called seasonal affective disorder (SAD), also known as winter depression. One explanation is the changing level of vitamin D3 during winter. One study, including 44 healthy subjects during winter, investigated the efficacy of vitamin D3 in enhancing mood. Results on a self-report showed that vitamin D3 significantly enhance positive affect [155].

Vitamin D has also been found to play an important role in brain development and function [156-159]. The study pointed out the wide distribution of vitamin D receptors throughout the brain. It showed that vitamin D has the ability to affect proteins which are known to be directly involved in learning and memory, motor control, and possibly even maternal and social behavior [160]. Maintaining vitamin D sufficiency in utero and during early life ensures the normal receptor transcriptional activity in the brain. This may be vital for brain development and also the maintenance of mental function later in life [161].

As shown in Table 3 the postulated efficacies of vitamin D, as demonstrated by the meta-analyses reported are largely unconvincing for most diseases/conditions apart from in relation to falls and fractures in the elderly [162-178]. Despite considerable efforts to explore the benefits of vitamin D, little convincing evidence has been presented in the form of meta-analyses in relation to the cardiovascular system, kidney function, Cystic fibrosis, diabetes, and mortality. The meta-analyses relied on disparate studies with varied approaches to measuring vitamin D analogues which may introduce significant questions regarding the results. It is suggested that further standardization should be introduced regarding which forms of vitamin D are measured and how they are measured.

## Conclusions

Vitamin D inadequacy is a global problem. Approximately 36% of otherwise healthy young adults and up to 57% of general medicine inpatients in the United States suffer from vitamin D inadequacy. These figures are even higher in Europe [71].

In the past, vitamin D intake was associated with the prevention of rickets in children whereas its effect in other areas has received little attention. In recent years, vitamin D deficiency has also been linked with the pathogenesis and/or progression of several disorders, including cancer, hypertension, multiple sclerosis, diabetes although the evidence for the associations of vitamin D with these conditions is generally weaker than it is for bone-related disease.

Although recommendations of daily vitamin D intake have been provided, higher levels are required in order to have real preventive or treatment effects as numerous studies have proved. UVB radiation plays an alternative in improving vitamin D content other than oral supplementation. Its advantage is that it will not cause vitamin D intoxication since excessive vitamin D will be broken down by UVB. However, a number of factors of the UVB such as wavelength, duration of exposure are needed to be carefully controlled so as to avoid erythema.

Despite the close link of vitamin D with human health, vitamin D inadequacy is not widely recognized as a problem by physicians and patients. Greater awareness of this problem is required among researchers, clinician, and patients of the high prevalence of vitamin D inadequacy.

## Competing interests

RZ was funded by a London Innovation Placements Programme sponsored by Allergy Matters Ltd (http://www.allergymatters.com).

## Authors' contributions

RZ conducted the initial substantial literature search, prepared the preliminary document and assisted with final version. DPN initiated the study, directed and augmented the literature search, and completed the final document. All authors have read and approved the final manuscript.
